# Examining the Role of Vitamin D in Caries Susceptibility in Children’s Deciduous Teeth: A Systematic Review

**DOI:** 10.3390/nu15224826

**Published:** 2023-11-18

**Authors:** Bogdan Andrei Bumbu, Magda Mihaela Luca, Roxana Buzatu

**Affiliations:** 1 Department of Dental Medicine, Faculty of Medicine and Pharmacy, University of Oradea, 410073 Oradea, Romania; bogdanbumbu@uoradea.ro; 2 Department of Pediatric Dentistry, Faculty of Dental Medicine, “Victor Babes” University of Medicine and Pharmacy Timisoara, Eftimie Murgu Square 2, 300041 Timisoara, Romania; 3 Department of Dental Aesthetics, Faculty of Dental Medicine, “Victor Babes” University of Medicine and Pharmacy Timisoara, Revolutiei Boulevard 9, 300041 Timisoara, Romania; roxana.buzatu@umft.ro

**Keywords:** vitamin D, dentistry, dental caries, food supplements

## Abstract

The global prevalence of dental caries in deciduous teeth remains a significant health concern, affecting almost 70% of children by the age of six in specific regions. This systematic review aspired to methodically investigate the association between vitamin D levels and susceptibility to caries in children’s deciduous teeth. A detailed search, guided by the PRISMA and PROSPERO guidelines, was conducted across three prominent electronic databases: PubMed, Web of Science, and Scopus, culminating in August 2023. The search integrated various keywords related to vitamin D and dental caries in primary dentition, yielding an initial pool of 1678 articles. After meticulous scrutiny, seven studies with a total of 7655 participants were deemed suitable for inclusion. The studies represented diverse geographical regions, showcasing varied vitamin D levels and sun exposure. Patient habits like brushing frequency, dental visits, and vitamin consumption also varied across studies. The analysis pinpointed vitamin D deficiency as a potential risk factor in some of the studies, with Odds Ratios (OR) ranging from 0.68 to 2.15. Statistically significant associations between vitamin D deficiency and caries susceptibility were documented in three studies (ORs of 2.15, 1.98, and 1.70). This comprehensive review elucidates the complex relationship between vitamin D levels and dental caries in children’s deciduous teeth. While some studies spotlight vitamin D’s pivotal role in dental health, inconsistencies across studies and regional differences necessitate more in-depth, globally representative investigations. Ensuring optimal vitamin D levels may play an integral role in dental health strategies. However, it is important to highlight that the roles of these studied factors might differ in deciduous teeth compared to permanent teeth.

## 1. Introduction

Dental caries, often recognized as a major public health issue, remains one of the most prevalent chronic diseases affecting children across the globe. As per the World Health Organization, dental caries in deciduous teeth affect 60–90% of school children [1,2,3]. This multifactorial disease not only results from the intricate interplay between host, microflora, substrate, nutrition, and dental hygiene, but is also influenced by systemic factors [4,5,6,7]. Despite significant advancements in preventive dentistry, the incidence of caries, especially in the delicate primary teeth, continues to be a substantial challenge [8].

Historically, vitamin D has been primarily recognized for its indispensable role in bone metabolism, calcium homeostasis, and the promotion of skeletal health [9,10]. Nonetheless, recent studies have illuminated that the scope of vitamin D’s physiological impact transcends its conventional association with calcium metabolism and bone remodeling, encompassing a broader range of physiological processes [11,12]. When examining the nexus between vitamin D and oral health, it becomes evident that vitamin D not only underpins tooth development and mineralization but also has profound implications for the equilibrium of the oral microbiome, the modulation of immune responses, and the optimization of salivary function [13,14,15]. These multifaceted roles of vitamin D, particularly its influence on the oral microbiome and immune response, can significantly modulate an individual’s predisposition to dental caries. Thus, delving deeper into this intricate relationship is of paramount importance for refining pediatric oral health strategies and interventions [16].

While often perceived as merely temporary placeholders, deciduous teeth serve vital functions that extend beyond the oral cavity, playing a critical role in maintaining space for the succeeding permanent teeth, significantly contributing to facial growth and aesthetics, and being essential for proper speech articulation and nutrition [17,18]. A compromised deciduous dentition, especially due to extensive caries, can adversely affect a child’s overall development, nutritional status, and even self-esteem, underscoring the importance of their preservation and care [19,20].

Recent studies have started to draw attention to the potential linkage between maternal vitamin D status during gestation and the onset of early childhood caries in deciduous teeth [21,22]. Thus, suboptimal maternal vitamin D levels during pregnancy were associated with an almost 50% higher risk of dental caries in children up to 2 years of age [22]. Such associations shed light on the idea that the foundation of robust oral health might be laid even before birth, emphasizing the importance of maternal nutrition and health during pregnancy [23,24]. This association, if further substantiated, could pave the way for novel preventive strategies targeting maternal health to ensure optimal oral health outcomes in offspring.

The staggering figures related to the global incidence of dental caries in deciduous teeth are a testament to the persistent nature of this challenge. In certain demographic segments and regions, statistics show that nearly 70% of children have encountered caries in their primary dentition by the age of six [25,26]. Such high prevalence rates necessitate an exhaustive understanding of all potential contributing factors, including systemic influencers like vitamin D, to frame effective intervention strategies. Given the intricate and multifactorial nature of dental caries and the potential influence of vitamin D, this systematic review aims to examine the available evidence linking serum vitamin D levels with caries predisposition in children’s deciduous (primary) teeth.

## 2. Materials and Methods

### 2.1. Protocol and Registration

The systematic review was carried out in August 2023 by evaluating three primary electronic databases: PubMed, Web of Science, and Scopus. We incorporated literature published through 2023. The search utilized medical subject headings (MeSH) keywords such as “Vitamin D”, “Calcitriol”, “Calcidiol”, “Cholecalciferol”, “25(OH)D”, “Primary Dentition”, “Dental Caries”, “Deciduous Teeth”, “Pediatric Oral Health”, “Dental Health”, “Primary Teeth”, and “Oral Health”. The search strategy was defined with the following string: “vitamin D” AND (“oral health” OR “primary teeth” OR “tooth diseases” OR “pediatric dentistry” OR “dental caries” OR “deciduous teeth” OR “primary dentition”).

Our review closely followed the Preferred Reporting Items for Systematic Reviews and Meta-Analyses (PRISMA) guidelines [27] and the International Prospective Register of Systematic Reviews (PROSPERO) criteria [28]. The aim was to methodically identify scientific articles exploring the relationship between vitamin D levels and caries susceptibility in children’s deciduous teeth. This systematic review was registered on the Open Science Framework (OSF) platform, using the registration code (osf.io/64wba).

### 2.2. Eligibility Criteria

Only English-language journal articles published up to 2023 were considered for inclusion. This ten-year timeframe was strategically chosen to encapsulate contemporary research developments in the domain of vitamin D and pediatric dental health. Initial stages of the selection process involved eradicating duplicate entries, followed by an in-depth evaluation of each abstract by a pair of independent researchers for pertinence to the study questions. Cross-referencing was conducted using the bibliographies of selected full-text publications. A meticulous examination of the remaining articles ensured alignment with the inclusion criteria.

For our systematic review, the inclusion criteria encompassed: (1) studies highlighting the correlation between vitamin D levels and caries susceptibility in children’s deciduous teeth; (2) clinical outcome measures inclusive of dental health, oral health, caries prevalence, and dental caries risk factors; (3) explicit methodologies detailing vitamin D status assessment; and (4) no time limit was set for publication date. Conversely, the exclusion criteria included: (1) studies not directly addressing the role of vitamin D on caries susceptibility in deciduous teeth; (2) studies devoid of relevant clinical outcomes data; (3) those not detailing vitamin D assessment methodologies; and (4) in vitro studies, case reports, conference proceedings, general reviews, commentaries, and editorial letters.

### 2.3. Data Collection Process

**Figure 1:**
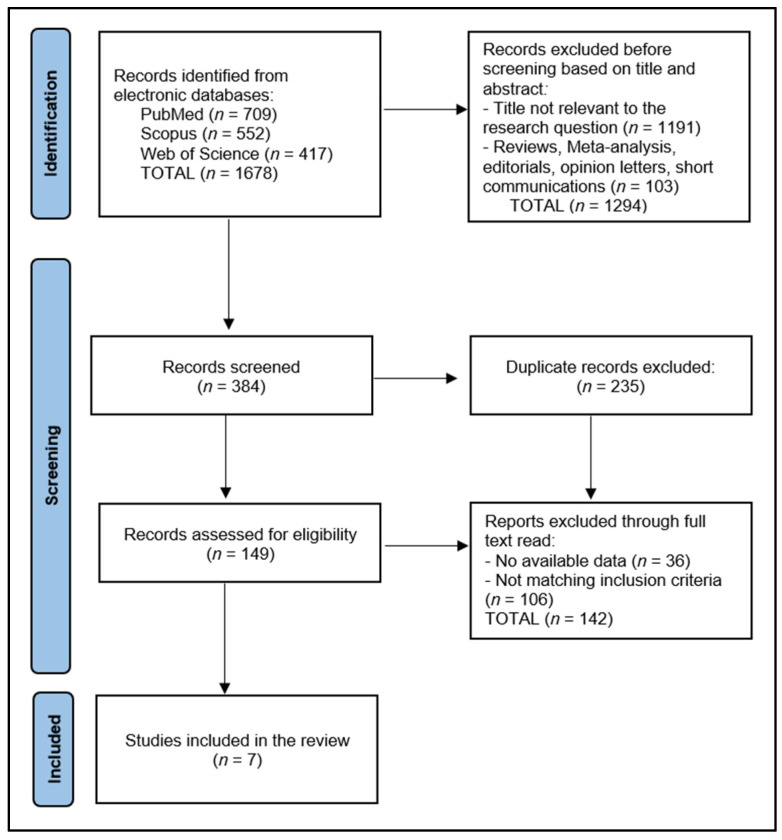
PRISMA Flow Diagram.

From the initial search, a total of 1678 articles were extracted, with a fraction of 235 identified as duplicates. A total of 7 studies were eligible for inclusion in the final analysis, as seen in Figure 1. Following the removal of non-pertinent articles based on abstracts, a rigorous screening of the remaining full-text articles was performed by two authors, with a third author engaged for validation. Ultimately, seven articles were deemed appropriate for inclusion in this review. Two researchers (M.M.L. and R.B) were involved in the screening and data extraction process, while a third researcher (B.A.B.) resolved any discrepancies.

The Quality Assessment Tool for Observational Cohort and Cross-Sectional Studies was employed to assess the chosen articles [29]. Each tool question was scored as 1 for “Yes” responses and 0 for “No” or “Other” responses, helping determine the cumulative performance score. Studies with scores ranging from 0 to 4 were categorized as poor quality, scores between 5 and 9 as fair quality, and those scoring 10 or above were considered of excellent quality. For transparency and robustness, two researchers (M.M.L. and R.B.) independently evaluated the quality of the selected studies.

### 2.4. Risk of Bias

**Figure 2:**
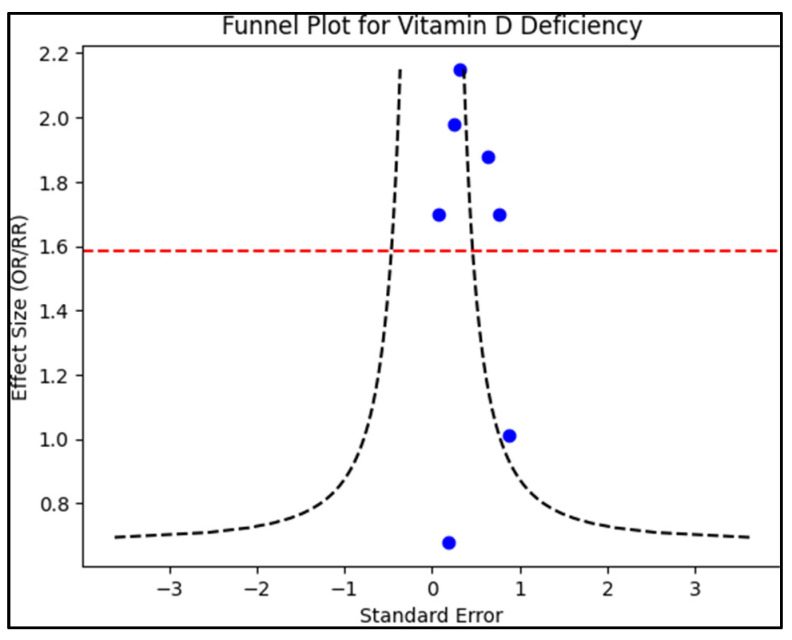
Funnel plot for publication bias.

A funnel plot was developed to assess publication bias, juxtaposing the standard error of the log odds ratio against its respective log odds ratio, as presented in Figure 2. The funnel plot’s symmetry was visually scrutinized and further verified using Egger’s regression test, where a *p*-value < 0.05 would denote significant publication bias. Additionally, a sensitivity analysis was conducted. This involved excluding individual studies sequentially and recalculating pooled odds ratios. The objective was to gauge the consistency of the results and discern the influence of singular studies on the overarching effect magnitude.

## 3. Results

### 3.1. Study Characteristics

The systematic review encompassed seven studies [30,31,32,33,34,35,36] that investigated various factors related to the development of dental caries in deciduous teeth, as detailed in Table 1. These studies spanned five countries: Canada, Germany, the USA, the Netherlands, and Egypt, demonstrating the widespread international focus on this subject. All studies were published from 2012 to 2023, indicating an ongoing and recent commitment to exploring these aspects of dental health. Specifically, amongst the studies published in 2023, only one study originated from Egypt [36]. The earlier studies were spread across Canada, Germany, the USA, and the Netherlands [30,31,32,33,34,35].

In terms of study design, the articles utilized a mixture of cross-sectional, cohort, and case–control approaches. Specifically, three studies were cross-sectional [30,32,36], three were cohort studies [31,34,35], and one was a case–control [33].

Upon evaluating the quality of these studies, three were rated as ‘Excellent’ [30,31,34], three as ‘Good’ [32,33,35], and one as ‘Fair’ [36]. The ‘Excellent’ quality studies were spearheaded by Schroth et al. [30], Wagner et al. [31], and Navarro et al. [34], two of which were cross-sectional and one cohort study. The ‘Good’ quality studies were conducted by Seminario et al. [32], Schroth et al. [33,35], representing both cross-sectional and case–control designs. The only study rated as ‘Fair’ was by El Shiekh et al. [36], which was cross-sectional in design, as seen in Table 1.

### 3.2. Children’s Characteristics

Table 2 provides a comprehensive overview of patient characteristics from the seven studies examining the role of vitamin D in caries susceptibility in children’s deciduous teeth. The cumulative number of participants from all studies reached a total of 7655. The smallest cohort was from Schroth et al. [35], with 38 participants, whereas the largest sample was observed in the study by Navarro et al. [34], which involved a number of 5257 individuals.

The patient groups in these studies predominantly consisted of two categories: those with insufficient vitamin D (25(OH)D) levels and those with normal vitamin D levels. The criteria for insufficiency were set at below 20 ng/mL or 50 nmol/L. Notably, the ratio of insufficient to normal vitamin D levels varied across studies. For instance, Schroth et al. [35] was skewed towards insufficiency with 32 out of 38 participants falling into this category, while Navarro et al.’s study [34] had an almost equal distribution.

The age range of the children under investigation spanned from infancy to 11 years old. The widest age range was observed in Seminario et al. [32] and Schroth et al. [35], both spanning 1–6 years, while Wagner et al. [31] and Navarro et al. [34] focused exclusively on younger children, with age ranges of 3–4 years and 6 years, respectively.

Geographically, the studies covered a broad latitudinal spectrum, from Egypt’s Cairo at 30° N to the expansive Canadian regions stretching up to 83° N. This wide geographic span indicates a diverse range of environmental influences, particularly concerning sun exposure and UV index. Indeed, the UV indices ranged from the low winter values of 0–1 in countries like Canada, Germany, and the Netherlands to extremely high summer values of 9–11+ in Cairo, Egypt.

The importance of understanding the impact of UV index becomes clear when considering the role of sun exposure in vitamin D synthesis. The studies from northern latitudes like Canada and Germany generally reported lower UV indices in the winter, suggesting potential seasonal influences on vitamin D levels. In stark contrast, El Shiekh et al.’s study from Cairo, Egypt [36], positioned closer to the equator, indicated higher UV indices year-round, which can have implications for vitamin D synthesis in the local population.

### 3.3. Children’s Habits

Table 3 provides a detailed examination of the habits of children across the seven studies, touching upon crucial determinants like brushing, hygiene practices, food choices, and supplement use, which can significantly influence oral health. The emphasis on oral hygiene, specifically tooth brushing, varied across studies. The vast majority of children in Wagner et al.’s study [31] brushed their teeth daily, reaching a high of 94.2%. In contrast, Schroth et al.’s 2012 study [35] reported a considerably lower proportion, with only 45.5% of children practicing daily brushing. It is essential to note that while some studies like Schroth et al. [30] provided specifics such as “2 times/day”, others like Wagner et al. [31] generalized with “daily brushing,” which might mask the exact frequency.

Dental visits, an indicator of dental hygiene awareness and access, also showed variability. While Schroth et al.’s 2016 study [30] showcased a percentage of 94.0% of children visiting the dentist at least annually, Wagner et al.’s study [31] reported a considerably lower percentage at 64.1%. Interestingly, this contrast suggests that while brushing habits were higher in Wagner et al.’s cohort, dental visits were less frequent compared to other studies.

Dietary habits, notably the consumption of milk, sugary drinks, or snacking patterns, were diverse. Schroth et al.’s 2016 study [30] reported equal percentages (64.0%) for daily milk and sugary drink consumption. El Shiekh et al.’s study [36] highlighted that over half the children indulged in snacking, which, depending on the nature of the snacks, could contribute to caries susceptibility.

Regarding supplement use, vitamin D supplementation displayed a strong prevalence in two studies (Schroth et al. [30] and Seminario et al. [32]) with 100% of the children taking it, possibly emphasizing its recognized importance in those cohorts. Fluoride supplementation also featured, but varied across studies, ranging from 37.3% in Schroth et al.’s 2016 study [30] to 63.3% in El Shiekh et al.’s study [36], suggesting varied perceptions or recommendations about fluoride use among the populations. Nevertheless, it is worth noting the gaps in some studies, such as Navarro et al.’s study [34] that had no information on hygiene, food habits, or supplement use, which might limit a comprehensive understanding of their cohort’s habits compared to others.

### 3.4. Risk Factors for Caries in Primary Teeth

Table 4 emphasizes the relationship between risk factors and caries susceptibility in primary teeth, focusing particularly on vitamin D deficiency and other associated behaviors. The impact of Vitamin D deficiency on caries risk is highlighted by several studies. Seminario et al.’s study [32] reported an Odds Ratio (OR) of 2.15 (CI: 1.61–2.86) and Schroth et al.’s 2015 study [33] an OR of 1.98 (CI: 1.55–2.53), both statistically significant and indicating an increased caries risk with Vitamin D deficiency. Navarro et al.’s study [34] also presented a significant association with an OR of 1.70 (CI: 1.57–1.85). In contrast, Schroth et al.’s 2016 study [30] showed an OR of 0.68 (CI: 0.40–1.14), not significant, suggesting a potential protective role of Vitamin D deficiency.

Regarding daily brushing, Wagner et al.’s study [31] and Schroth et al.’s 2016 study [30] indicated protective effects with ORs of 0.71 (CI: 0.38–1.32) and 0.51 (CI: 0.33–0.79), respectively, the latter being statistically significant. El Shiekh et al.’s study [36] showed a statistically significant negative OR of −2.21 (CI: −4.14–−0.28), which raises questions about the data interpretation.

Yearly dental visits, as captured in two studies, showed varied results. Schroth et al.’s 2016 study [30] suggested an increased caries risk with an OR of 1.90 (CI: 0.82–4.41), while Wagner et al.’s study [31] presented a higher and significant OR of 4.51 (CI: 3.82–5.34).

For snacking and sugary food consumption, Wagner et al.’s study [31] indicated a protective effect with a significant OR of 0.30 (CI: 0.10–0.92), while Schroth et al.’s 2016 study [30] and El Shiekh et al.’s study [36] showed ORs closer to 1, implying no strong association.

Finally, vitamin supplement intake varied across studies. Schroth et al.’s 2015 study [33] revealed a slightly increased caries risk with a decreased oral intake of vitamin D (OR: 1.51, CI: 0.81–2.82). Navarro et al.’s study [34] indicated a near-neutral association (OR: 0.96, CI: 0.95–0.97). Fluoride supplementation, reported in Schroth et al.’s 2016 study [30] and El Shiekh et al.’s study [36], showed ORs of 1.01 (CI: 0.58–1.75) and 2.19 (CI: −1.25–5.63), respectively, suggesting no clear trend in its role on caries risk.

## 4. Discussion

This systematic review presented an extensive overview of factors affecting the development of dental caries in deciduous teeth. Drawing from a wide geographical spread including Canada, Germany, the USA, the Netherlands, and Egypt, the results suggest a multi-faceted influence of various factors that spans across different cultures and environmental contexts.

In terms of vitamin D levels, the juxtaposition of studies like Schroth et al. [35] and Navarro et al. [34] demonstrates the heterogeneity in the population. The prominent categorization based on vitamin D insufficiency gives an insight into the probable significance of this vitamin in oral health. The selected cutoff for vitamin D insufficiency, below 20 ng/mL or 50 nmol/L, while standard, is still debated among researchers, and the study’s conclusions might be influenced by this threshold [37,38].

The geographical diversity of the studies, especially when evaluated against UV index and its relation to vitamin D synthesis, offers a nuanced understanding. For instance, Canada’s low UV indices, particularly during winter, might inherently predispose its population to lower vitamin D levels as compared to Cairo, Egypt with its consistently high UV indices. This distinction is crucial as vitamin D, synthesized predominantly through sun exposure, plays a vital role in bone and dental health [39]. Vitamin D’s positive impact on oral health extends beyond solely tooth mineralization; it also has anti-inflammatory properties and boosts the production of anti-microbial peptides [40].

Child habits, such as brushing frequency and dietary choices, showed considerable variation across the studies. The vast difference between the high brushing frequency reported by Wagner et al. [31] and the relatively lower frequency from Schroth et al.’s 2012 study [35] might suggest regional, educational, or cultural influences. Moreover, the contrasting dental visit frequencies between the two aforementioned studies emphasize the difference in dental awareness or accessibility among populations.

The association between vitamin D deficiency and caries susceptibility emerged as a salient point. While most studies suggested an increased caries risk with vitamin D deficiency, the divergent results, such as those from Schroth et al.’s 2016 study [30], accentuate the complex interplay of factors and the possible influence of confounders. Similarly, oral hygiene habits and dietary choices, along with supplement intake, underscore the multifactorial nature of dental caries. For instance, the counter-intuitive protective effect of sugary snacks in Wagner et al.’s study [31] suggests that there may be underlying factors, not captured in the study, which influence the outcome.

In a recent study it was found that individuals with insufficient levels of vitamin D are at a 22% higher risk of developing dental caries compared to their counterparts with adequate vitamin D [41]. Particularly noteworthy is that children with a vitamin D deficiency showed an almost 70% higher risk of dental caries in their deciduous teeth, compared to children with permanent teeth. However, our systematic review did not evaluate those with permanent teeth to avoid any confounding factors regarding primary and secondary dentition differences. This heightened risk in deciduous teeth might stem from the lower mineralization characteristic of deciduous teeth and the challenges younger children face in maintaining oral hygiene [42]. Similar findings regarding the dental caries in children also found significantly higher prevalences of caries in deciduous teeth compared to permanent teeth [43]. Additionally, the pronounced immune regulatory and antibacterial properties of vitamin D, especially against cariogenic bacteria, seem more evident in this pediatric population.

It is also worth discussing the methods utilized across the different studies to measure dental caries experience varied, including metrics like prevalence, Decayed, Missing, and Filled Teeth (DMFT)/Decayed, Missing, and Filled Surfaces (DMFS) indices, or even subsets of these indices [44,45,46]. Such disparities in measurement approaches could lead to inconsistent results. The definitions of caries were not consistent across studies either; some included only cavitated lesions while others factored in non-cavitated lesions. Moreover, many studies remain ambiguous about the cut-off points used in defining caries, with or without objective or subjective measurements [47].

Several limitations within this systematic review merit consideration. First, the review was restricted to English-language publications, potentially leading to the omission of relevant research conducted in other languages. The span of a decade (2013–2023) for the included research, though comprehensive, might miss key studies or emerging trends immediately before or after this period. While the review sourced articles from three major electronic databases, other databases might have been overlooked. Given the varying study designs (cross-sectional, cohort, and case–control) of the included studies, the synthesis of evidence presents challenges, especially when cross-comparing findings. The quality of incorporated studies ranged from ‘Excellent’ to ‘Fair’, meaning some studies’ results might have greater susceptibility to bias or methodological issues than others. Furthermore, the significant range in study sizes, from as few as 38 participants to as many as 5257, could influence the generalizability of findings. The funnel plot and sensitivity analysis provided insights into potential biases, but residual confounding and bias inherent in observational studies cannot be fully eradicated. Moreover, it is important to highlight that the roles of these studied factors might differ in deciduous teeth compared to permanent teeth, that were not studied in the current systematic review to avoid the confounding effect of different teeth structures. Lastly, the variability in methods used across studies to assess vitamin D levels and caries might have introduced heterogeneity in outcomes, making it challenging to draw robust conclusions.

## 5. Conclusions

Several studies conducted across diverse geographical locations with varying levels of sun exposure indicated that there is a probable association between vitamin D deficiency and an increased risk for caries in deciduous teeth, with certain studies reporting statistically significant odds ratios. However, this relationship was not consistent across all included studies. Notably, other risk factors such as daily brushing habits and annual dental visits displayed varying degrees of association with caries, emphasizing the multifactorial nature of dental caries in children. Nevertheless, it is worth noting that deciduous teeth might be impacted differently by these studied factors, as compared to permanent teeth.

## Figures and Tables

**Table 1:** Study characteristics.

Study & Author		Country	Study Year	Study Design	Study Quality
1	Schroth et al. [30]	Canada	2016	Cross-sectional	Excellent
2	Wagner et al. [31]	Germany	2016	Cohort	Excellent
3	Seminario et al. [32]	USA	2018	Cross-sectional	Good
4	Schroth et al. [33]	Canada	2013	Case-control	Good
5	Navarro et al. [34]	Netherlands	2021	Cohort	Excellent
6	Schroth et al. [35]	Canada	2012	Cohort	Good
7	El Shiekh et al. [36]	Egypt	2023	Cross-sectional	Fair

**Table 2:** Patients’ background characteristics and regional sun exposure.

Study Number		Number of Participants	Study Groups (Vitamin D Levels)	Age	Region (Latitude Range)	Sun Exposure (UV Index)
1	Schroth et al. 2016 [30]	1017	Insufficient (127) vs. Normal (890)	6–11 years	Manitoba—Canada (49° N–60° N)	Winter: UV 0–1 (Low), Summer: UV 4–7 (Moderate to High)
2	Wagner et al. 2016 [31]	755	Insufficient (60) vs. Normal (695)	3–4 years	Thuringia—Germany (50° N–52° N)	Winter: UV 0–1 (Low), Summer: UV 5–7 (Moderate to High)
3	Seminario et al. 2018 [32]	276	Insufficient (138) vs. Normal (138)	1–6 years	Washington—USA (45° N–49° N)	Winter: UV 0–2 (Low), Summer: UV 5–8 (Moderate to Very High)
4	Schroth et al. 2013 [33]	261	Insufficient (43) vs. Normal (218)	3–5 years	Manitoba—Canada (49° N–60° N)	Winter: UV 0–1 (Low), Summer: UV 4–7 (Moderate to High)
5	Navarro et al. 2021 [34]	5257	Insufficient (2620) vs. Normal (2637)	6 years	South Holland—Netherlands (51° N–52° N)	Winter: UV 0–1 (Low), Summer: UV 5–7 (Moderate to High)
6	Schroth et al. 2012 [35]	38	Insufficient (32) vs. Normal (6)	1–6 years	All regions—Canada (41° N–83° N)	Varies widely
7	El Shiekh et al. 2023 [36]	51	Insufficient (34) vs. Normal (17)	3–5 years	Cairo—Egypt (30° N)	Winter: UV 3–5 (Moderate), Summer: UV 9–11+ (Very High to Extreme)

Vitamin D (25(OH)D) hypovitaminosis (insufficiency) is considered below 20 ng/mL or 50 nmol/L; vitamin D deficiency is considered below 10 ng/mL or 25–30 nmol/L; UV—Ultraviolet light.

**Table 3:** Children’s brushing, hygiene, food, and vitamin D supplementation habits.

Study Number		Brushing	Hygiene	Food	Supplements
1	Schroth et al. [30]	2 times/day—70.9%	Visits the dentist at least once a year—94.0%	Drink milk 1/day—64.0%; Sugary drinks 1/day—64.0%	Vitamin D—100% Fluoride—37.3%
2	Wagner et al. [31]	Daily brushing—94.2%	Visits the dentist at least once a year—64.1%	Sugary drinks/food > 3 times daily—2.3%	Vitamin D—92.1%
3	Seminario et al. [32]	NR	NR	NR	Vitamin D—100%
4	Schroth et al. [33]	1–2 times/day	Visits the dentist at least once a year	Breast-fed—78.2%	Multivitamins—54.8%
5	Navarro et al. [34]	≥2 times/day—51.2%	NR	NR	NR
6	Schroth et al. [35]	Daily brushing—45.5%	Visits the dentist at least once a year—46.7%	Drink milk 1/day—50.0%	Multivitamins—44.4%
7	El Shiekh et al. [36]	Daily brushing—60.9%	Visits the dentist at least once a year—78.5%	Snacking—53.5%	Fluoride—63.3%

NR—Not Reported.

**Table 4:** Risk factors for caries in primary teeth.

Risk Factors (OR/RR)		Vitamin D Deficiency	Daily Brushing	Yearly Dental Visits	Snacking/Sugary Foods	Vitamin Supplements	Adjusted Factors in Analysis
1	Schroth et al. [30]	0.68 (0.40–1.14)	0.51 * (0.33–0.79)	1.90 (0.82–4.41)	1.09 (0.78–1.54)	Fluoride 1.01 (0.58–1.75)	Daily brushing, Yearly dental visits, Snacking
2	Wagner et al. [31]	1.88 (0.99–3.51)	0.71 (0.38–1.32)	4.51 (3.82–5.34)	0.30 (0.10–0.92)	NR	Daily brushing, Yearly dental visits
3	Seminario et al. [32]	2.15 * (1.61–2.86)	NR	NR	NR	NR	None (Unadjusted)
4	Schroth et al. [33]	1.98 * (1.55–2.53)	0.97 (0.94–1.01)	NR	NR	Vitamin D drops 1.51 (0.81–2.82)	Daily brushing
5	Navarro et al. [34]	1.70 * (1.57–1.85)	NR	NR	NR	0.96 (0.95–0.97)	None (Unadjusted)
6	Schroth et al. [35]	1.70 (0.75–3.83)	0.21 (NR)	NR	NR	0.65 (NR)	Daily brushing
7	El Shiekh et al. [36]	1.01 (−0.74–2.76)	−2.21 * (−4.14–−0.28)	−1.43 (−3.09–−0.23)	−1.18 (2.42–0.89)	Fluoride drops/tablets 2.19 (−1.25–5.63)	Daily brushing, Yearly dental visits, Snacking

Vitamin D refers to 25(OH)D levels; *—statistically significant values; NR—Not Reported; OR—Odds Ratio; RR—Risk Ratio.

## Data Availability

Not applicable.
